# Bilateral Congenital Posterior Hemivertebrae and Lumbar Spinal Stenosis Treated With Posterior Spinal Fusion and Instrumentation

**DOI:** 10.5435/JAAOSGlobal-D-19-00054

**Published:** 2019-10-02

**Authors:** Alexander Nazareth, Lindsay M. Andras, Mark D. Krieger, David L. Skaggs

**Affiliations:** From the Keck School of Medicine, University of Southern California, Los Angeles, CA (Dr. Nazareth, Dr. Andras, Dr. Krieger, and Dr. Skaggs); the Children's Orthopaedic Center, Children's Hospital Los Angeles, Los Angeles, CA (Dr. Nazareth, Dr. Andras, and Dr. Skaggs); and the Department of Neurosurgery, Children's Hospital Los Angeles, Los Angeles, CA (Dr. Krieger).

## Abstract

Posterior hemivertebrae are wedge shaped deformities that can result in progressive kyphosis. Surgical intervention at an early age may be required, however choice of surgical technique is controversial. The aim of this report was to describe a case of progressive congenital lumbar kyphosis and bilateral posterior hemivertebra with retropulsion of tissue into the spinal canal treated successfully by posterior spinal fusion and instrumentation without anterior hemivertebra resection or decompression. We report on a patient with bilateral lumbar posterior hemivertebra at L1-L2 treated with posterior spinal fusion and instrumentation at less than 1 year of age. At 10 mo of age, the patient underwent posterior spinal fusion and instrumentation with resection of L1 and L2 posterior elements. No resection of the anterior aspect of the bilateral hemivertebrae was performed. Correction of the kyphotic deformity was maintained at last radiographic follow-up at five years post-operatively and there is no evidence of spinal stenosis. Early intervention with resection of posterior elements and fusion with instrumentation for bilateral congenital lumbar hemivertebrae provided adequate deformity correction and maintenance of the spinal canal width without anterior resection. Despite his young age, instrumentation was both feasible and beneficial in maintaining alignment.

Posterior hemivertebrae are wedge-shaped deformities caused by failure of formation of the anterior part of the vertebra that result in progressive kyphosis.^1^ Previously described surgical procedures include in situ posterior or AP fusion with or without instrumentation and hemivertebra excision with fusion.^2,3,4^

## Case Report

**Figure 1:**
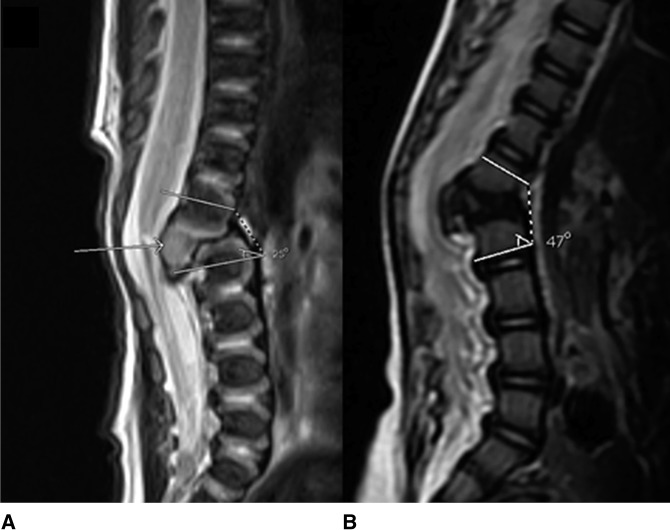
**A**, MRI at day 2 of life shows notable canal intrusion (arrow) and 25° local kyphosis. **B**, MRI at 9 months of age shows 47° of local kyphosis. Both are done supine, under anesthesia. (Reproduced with permission from the Children's Orthopaedic Center, Los Angeles, CA.)

**Figure 2:**
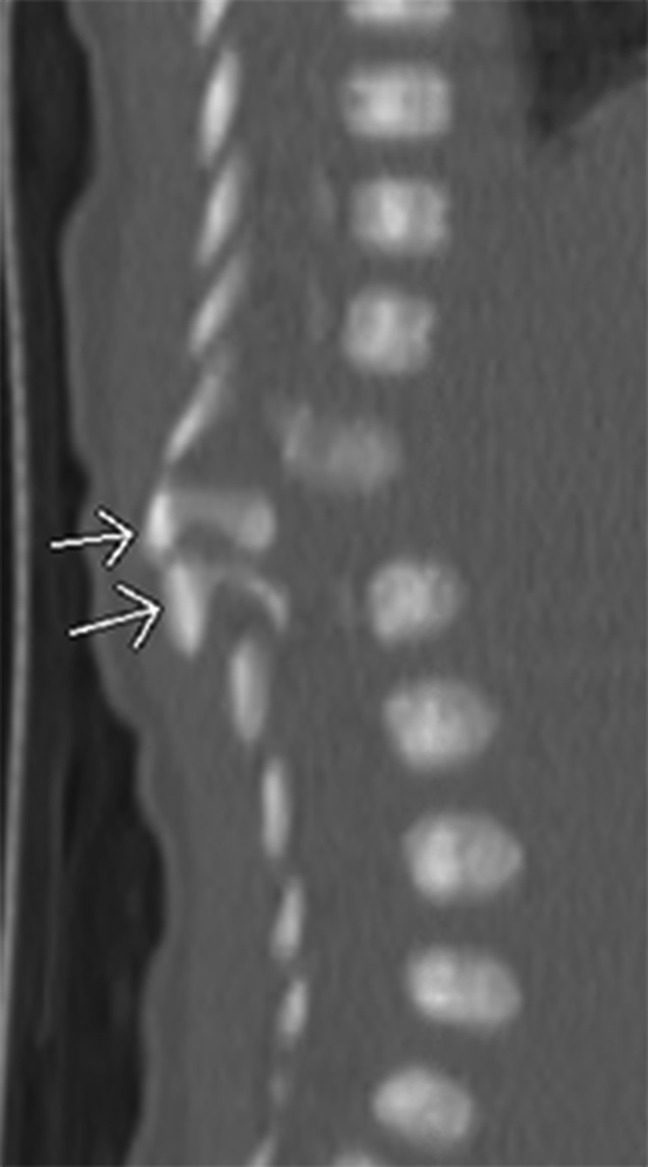
Sagittal CT scan taken at day 2 of life (at an outside institution) demonstrates two posterior hemivertebrae, with osseous posterior elements visualized without a corresponding vertebral body anteriorly. (Reproduced with permission from the Children's Orthopaedic Center, Los Angeles, CA.)

**Figure 3:**
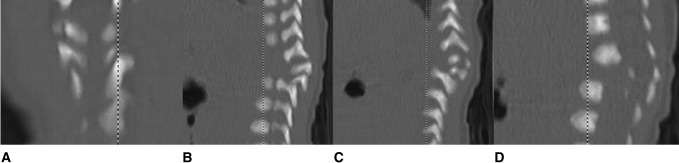
Various CT cuts demonstrating the bilaterial posterior hemivertebrae (A-D). (Reproduced with permission from the Children’s Orthopaedic Center, Los Angeles, CA)

The patient is the product of a pregnancy complicated by intrauterine growth restriction; otherwise, birth and family histories were normal. Bilateral lumbar posterior hemivertebrae were diagnosed by ultrasonography in utero. CT and MRI (Figures 1–3) were done at an outside institution on day 2 of life. He was meeting all developmental and growth milestones without any neurological signs or symptoms.

He presented to our institution at 6 months of age and was initially placed in a thoracolumbosacral orthosis. Follow-up MRI at 9 months of age (Figure 1, B) showed progressive worsening of his thoracolumbar kyphosis from 25° at birth to 47° at 9 months. Note that both MRIs were done in a supine position. Clinically, the deformity was also noted to be progressive. The growth plates of the lumbar hemivertebrae were angulated in a manner that was concerning for possible spinal canal stenosis and worsening kyphosis with additional growth. Additional observation and worsening of the deformity could be harmful. In addition to deciding on whether to proceed with surgery at this time, the ability to provide adequate fixation with pedicle screws in a patient that was less than a year old with 2.5-mm pedicles was a concern. Consideration was also given to whether removal of the abnormal bone anteriorly that was narrowing the canal was necessary.

**Figure 4:**
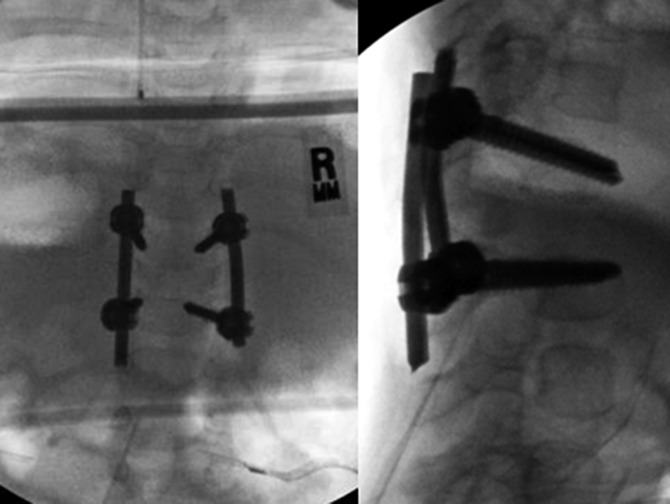
Fluoroscopic imaging AP and lateral demonstrating T12-L3 posterior spinal fusion and instrumentation. Fifteen degrees of kyphosis correction by compressing the screws on the rods. Screws were placed so they were just barely bicortical to provide additional stability given his small size and age. (Reproduced with permission from the Children's Orthopaedic Center, Los Angeles, CA.)

After discussion with the family as well as both our orthopaedic and neurosurgical teams, the patient underwent posterior instrumentation and fusion from T12-L3. Resection of the posterior elements of the abnormal hemivertebrae at L1 and L2 was removed. No attempt at resection of the anterior elements or circumferential exposure of the spinal cord was done. The pedicle screws were intentionally long, so that they were just barely bicortical to provide more robust fixation. Approximately 15° of kyphosis correction by compressing the screws on the rods was found. (Figure 4).

**Figure 5:**
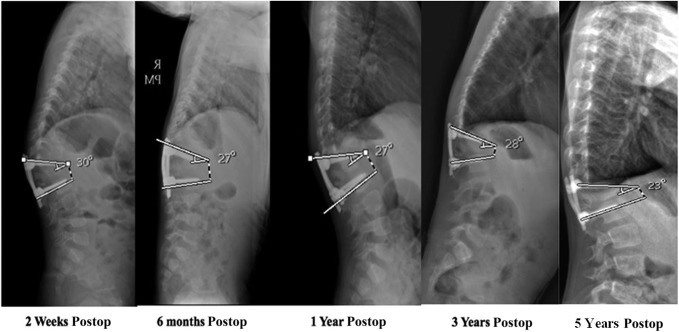
Lateral plain radiograph imaging demonstrating sustained kyphotic deformity correction through 5 years postoperatively. (Reproduced with permission from the Children's Orthopaedic Center, Los Angeles.)

**Figure 6:**
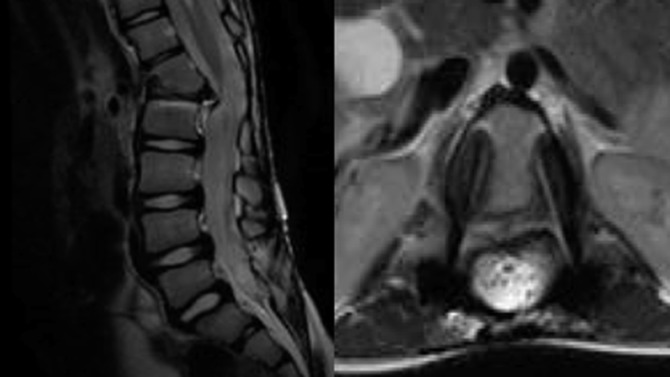
MRI at last follow-up (5 years postoperatively) demonstrating adequate canal space. (Reproduced with permission from the Children's Orthopaedic Center, Los Angeles.)

**Figure 7:**
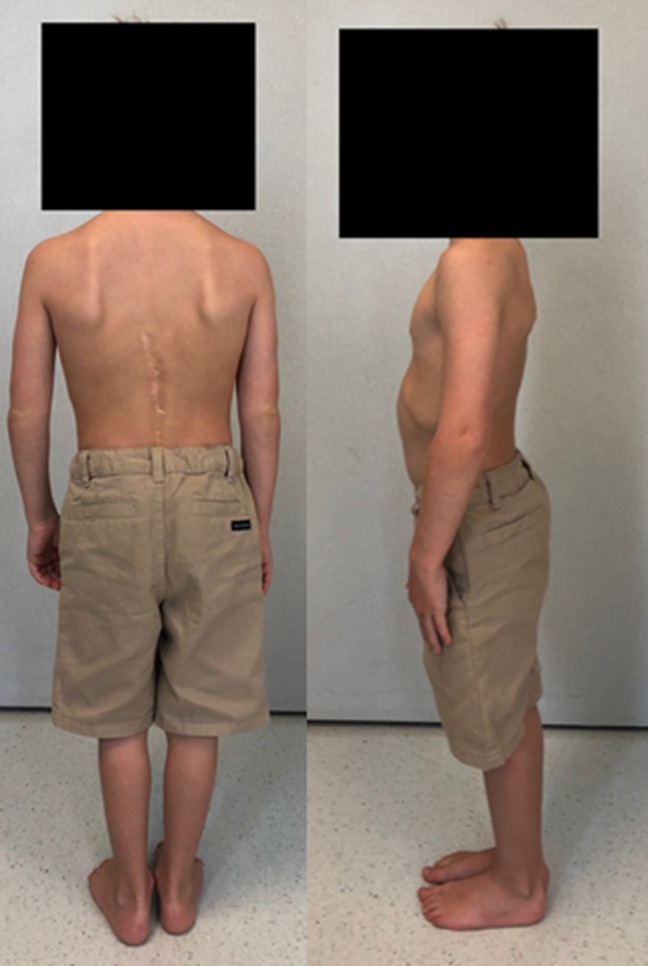
Clinical images at last follow-up (5 years postoperatively). (Reproduced with permission from the Children's Orthopaedic Center, Los Angeles.)

Correction of the kyphotic deformity was maintained at last radiographic follow-up at 5 years postoperatively with approximately 23° of localized kyphosis seen on plain radiograph (Figures 5 and 6). Physical examination at time of last follow-up is perhaps even more impressive, showing a clinically straight back without any appreciable kyphosis in the thoracolumbar region (Figure 7). The patient currently lives an active lifestyle without activity restrictions and takes karate lessons in addition to recreationally swimming and working out. Per his father's report, he is able to do “200 pushups in under 5 minutes.”

## Discussion

We describe a case of congenital lumbar kyphosis due to two bilateral posterior hemivertebrae at L1 and L2 with retropulsion of the vertebral body into the spinal canal treated successfully by posterior spinal fusion without resection of the anterior aspect of the hemivertebra. Sagittal plane correction and normalization of lumbar spinal canal width were observed and maintained at the most recent follow-up at approximately 5 years postoperatively.

Treatment of congenital kyphosis secondary to posterior hemivertebrae should aim to obtain a balanced spine and prevent progression of the deformity. Although variable, these deformities are associated with an average progression of 7° per year, and surgical treatment is associated with good outcomes.^5,6,7^ Timing of surgery depends on the age of the patient, severity and progression of kyphosis, and the presence of neurologic symptoms. Winter et al.^1^ reported on the clinical management and natural history of congenital kyphosis in a retrospective study of 130 patients. Their treatment algorithm consisted of early posterior fusion for patients younger than 3 years with mild deformities and combined AP fusion in deformities with a kyphotic angle of more than 50°. However, both timing of surgery and technique remain controversial in the management of hemivertebrae. Surgeon experience and comfort with these management options is also an important consideration. An alternative treatment for this issue would have been an anterior resection with posterior stabilization, which was contemplated. Concerns were found however with doing an open anterior approach on a child of such a young age, as well as concerns about inability to control the deformity between the anterior resection and posterior stabilization. Particularly, given the nature of this deformity, one could imagine that after the anterior resection, the cord could drift anteriorly, and this may not be well tolerated putting the child at more neurologic risk. With an all posterior approach without a resection, we were able to gradually correct the deformity with greater control which we felt was a notable advantage that outweighed the benefit of complete correction of the kyphosis with a combined anterior and posterior approach. Given the current radiographic and clinical appearance, we feel that the small residual kyphosis created little if any negative consequence.

The advantage of early surgical intervention and obtaining posterior fusion are the prevention of an increasing deformity due to anterior growth at the apex of the deformity and subsequent neurological sequelae.^8^ Chang et al.^9^ in a retrospective study of children with hemivertebrae found patients treated before the age of 6 years had a markedly better deformity correction without a negative effect on vertebral body or spinal canal growth. We do not recommend posterior spinal fusion and instrumentation alone as standard practice for all patients with dorsal hemivertebrae and resultant spinal stenosis. Combined AP fusion with decompression should be the preferred surgical management in patients with severe kyphosis or neurologic compromise.^10,11^ However, short segmental fusion can be done effectively and safely on very young children and therefore warrants consideration. For patients with a moderate kyphotic deformity (<50°), the morbidity of an anterior approach outweighs the possibly greater correction of kyphosis with a combined AP approach. Although the pedicles were small, we were able to safely place screws using traditional landmarks and verification with fluoroscopy. In this case, the pedicle screws were placed, so that they were just bicortical. The screws that were placed were 3.5 × 24 mm and 3.5 × 26 mm. Although these screws were small, we feel they allowed for superior fixation over alternatives such as hooks or sublaminar wires alone. In small children, a high level of concern regarding the potential for screws to cutout during the correction should always be there as the bone is much softer than in older age groups. A technical tip is to try to obtain as much correction as possible before compression on the screws and final construct itself. The following can often be achieved by indirect reduction from pressure on the patient's body/trunk. In addition, a third rod on either the ribs or spine can often be a beneficial reduction tool. This rod can be placed with hooks that may plow during the correction but can subsequently be removed once the correction is achieved and the “final” rods are placed in the screws and secured.

Although this rod provided additional stability in small, weak bone, they are just barely bicortical to avoid potentially devastating vascular complications. This aspect of the technique is highly reliant on surgeon experience, and this aspect is preferable to err on the shorter side if any doubt about the length and safety of screw position is present.

This case report presents a less morbid, alternative surgical technique to complete hemivertebra resection in infants with lumbar posterior hemivertebrae and spinal stenosis. We hope to provide clinicians with an additional tool in the surgical management of the congenital hemivertebra in infants with a progressive moderate kyphotic deformity (<50°) and intact neurological function.
